# Cost-effectiveness of diagnostic for malaria in Extra-Amazon Region, Brazil

**DOI:** 10.1186/1475-2875-11-390

**Published:** 2012-11-23

**Authors:** Maria Regina F de Oliveira, Silvana P Giozza, Henry M Peixoto, Gustavo AS Romero

**Affiliations:** 1 School of Medicine, University of Brasília, Brasilia, Brazil; 2 National Institute for Science and Technology for Health Technology Assessment (IATS/CNPq), Porto Alegre, Rio Grande do Sul, Brazil; 3 Ministry of Health, Brasilia, Brazil; 4 State Secretariat of Health, Brasilia, Brazil

**Keywords:** Malaria, Diagnoses, Rapid test, Microscopy, Economic evaluation

## Abstract

**Background:**

Rapid diagnostic tests (RDT) for malaria have been demonstrated to be effective and they should replace microscopy in certain areas.

**Method:**

The cost-effectiveness of five RDT and thick smear microscopy was estimated and compared. Data were collected on Brazilian Extra-Amazon Region. Data sources included the National Malaria Control Programme of the Ministry of Health, the National Healthcare System reimbursement table, laboratory suppliers and scientific literature. The perspective was that of the Brazilian public health system, the analytical horizon was from the start of fever until the diagnostic results provided to patient and the temporal reference was that of year 2010. Two costing methods were produced, based on exclusive-use microscopy or shared-use microscopy. The results were expressed in costs per adequately diagnosed cases in 2010 U.S. dollars. One-way sensitivity analysis was performed considering key model parameters.

**Results:**

In the cost-effectiveness analysis with exclusive-use microscopy, the RDT CareStart™ was the most cost-effective diagnostic strategy. Microscopy was the most expensive and most effective, with an additional case adequately diagnosed by microscopy costing US$ 35,550.00 in relation to CareStart™. In opposite, in the cost-effectiveness analysis with shared-use microscopy, the thick smear was extremely cost-effective. Introducing into the analytic model with shared-use microscopy a probability for individual access to the diagnosis, assuming a probability of 100% of access for a public health system user to any RDT and, hypothetically, of 85% of access to microscopy, this test saw its effectiveness reduced and was dominated by the RDT CareStart™.

**Conclusion:**

The analysis of cost-effectiveness of malaria diagnosis technologies in the Brazilian Extra-Amazon Region depends on the exclusive or shared use of the microscopy. Following the assumptions of this study, shared-use microscopy would be the most cost-effective strategy of the six technologies evaluated. However, if used exclusively for diagnosing malaria, microscopy would be the worst use of resources. Microscopy would not be the most cost-effective strategy, even when structure is shared with other programmes, when the probability of a patient having access to it was reduced. Under these circumstances, the RDT CareStart™ would be the most cost-effective strategy.

## Background

Brazil presents two quite distinct epidemiological situations with regard to malaria transmission. The Amazon Region – which includes nine states of the federation – is an area with high endemicity that annually registers 99.8% of the country’s malaria cases, with an Annual Parasitic Incidence (API) of more than 49.9 cases per 1,000 inhabitants in some transmission areas [1-3]. The area known as the Extra-Amazon Region – composed of 17 states and the Federal District – registers more than 1,000 new cases per year, including imported and autochthonous ones [4]. In this area, in the year 2010, 1,263 cases of malaria were registered, with 901 (71%) of them caused by *Plasmodium vivax*[4]. The states of São Paulo (SP), Espírito Santo (ES), Minas Gerais (MG), Goiás (GO) and the Federal District (DF) notified 50% of the cases registered in 2010 in the whole Extra-Amazon Region; the cases dealt with in this area are mostly imported from Amazonia or from countries with malaria transmission, such as African countries and Paraguay, but there are some autochthonous cases, mainly from São Paulo and Espírito Santo states [4].

Due to the low incidence of malaria in the Extra-Amazon Region, it is a great challenge to diagnose it, requiring doctors trained in diagnosis of suspected cases and in the opportune treatment of these, as well as laboratories ready to make early and accurate specific diagnostic. As the Extra-Amazon area receives imported cases, especially from African countries, opportune diagnostic is essential for the appropriate management of the disease, in order to prevent severe manifestations of malaria and deaths caused, especially, by *Plasmodium falciparum*.

Microscopy by the thick smear technique is the most widely used diagnostic method, including in the Extra-Amazon area. It is a low-cost test that, however, demands experienced professionals to carry it out and read the results. Variations in the execution technique and loss of slide quality can compromise the accuracy of the test, which is considered the gold standard for malaria [5]. In the Extra-Amazon Region, diagnosis by microscopy is carried out in centers of reference for the test, situated in all the state capitals and in towns in areas where there have, historically, been more cases recorded. Suspected cases are sent to these reference centers for diagnosis, and these centers are sometimes not where the patients live. To have easy access to diagnostic of malaria is a relevant factor for the economic costs of the disease. Macaluley [6] studied a strategy of aggressive active case detection; despite being more expensive, this strategy potentially can be clearly worthwhile, especially because of the diagnostic of asymptomatic malaria and the expanded access to diagnosis in populations living in areas of malaria transmission risk. Pang [7] studied a community-based programme incorporating dipstick tests for malaria management, which improved the access to diagnosis in remote areas, concluding that it could have economic advantages.

Rapid Immunochromatographic Diagnostic Tests (RDTs) to diagnose malaria were developed in the 1990s. These are tests that detect *Plasmodium* antigens in the peripheral blood, by means of a finger-prick, and furnish the diagnosis in 15 minutes. They do not require laboratory structure or highly experienced professionals for their execution [8], but their high cost compared to the thick smear test is one of their drawbacks for use on a large scale. RDTs have been recommended by the World Health Organization (WHO) and by the Brazilian Ministry of Health for use in remote areas [8-10], where there are no laboratories for microscopy available; in Brazil, RDTs are also recommended for use in the Extra-Amazon area [10].

The objective of this study was to estimate the incremental cost-effectiveness ratio, considering the use of five commercial RDT brands for malaria, compared with the conventional diagnosis method by thick smear, for the year 2010. This is the first cost-effectiveness analysis for malaria diagnosis in the Extra-Amazon Region and the first that evaluates five different commercial tests in Brazil. The information will be a help to decision-makers with respect to the use of RDTs in the Region.

## Methods

### Diagnostic strategies evaluated

Five RDTs for diagnostic of new cases of malaria due to *P. falciparum* and *P. vivax* were evaluated in comparison to the conventional diagnostic strategy – thick smear microscopy. The RDTs evaluated were: 1) SD Bioline FK60 (PF/Pan)™ – Bioline; 2) CareStart (Pan)™ - DiaSys; 3) First Response Malaria Combo™ - Premier Medical Corporation Ltd.; 4) Parascreen™ (Pf/Pan) – Zephyr Biomedicals by The Tulip Group; and 5) ICT BinaxNOW Malaria™ - BinaxNOW.

### Decision analytic model

**Figure 1:**
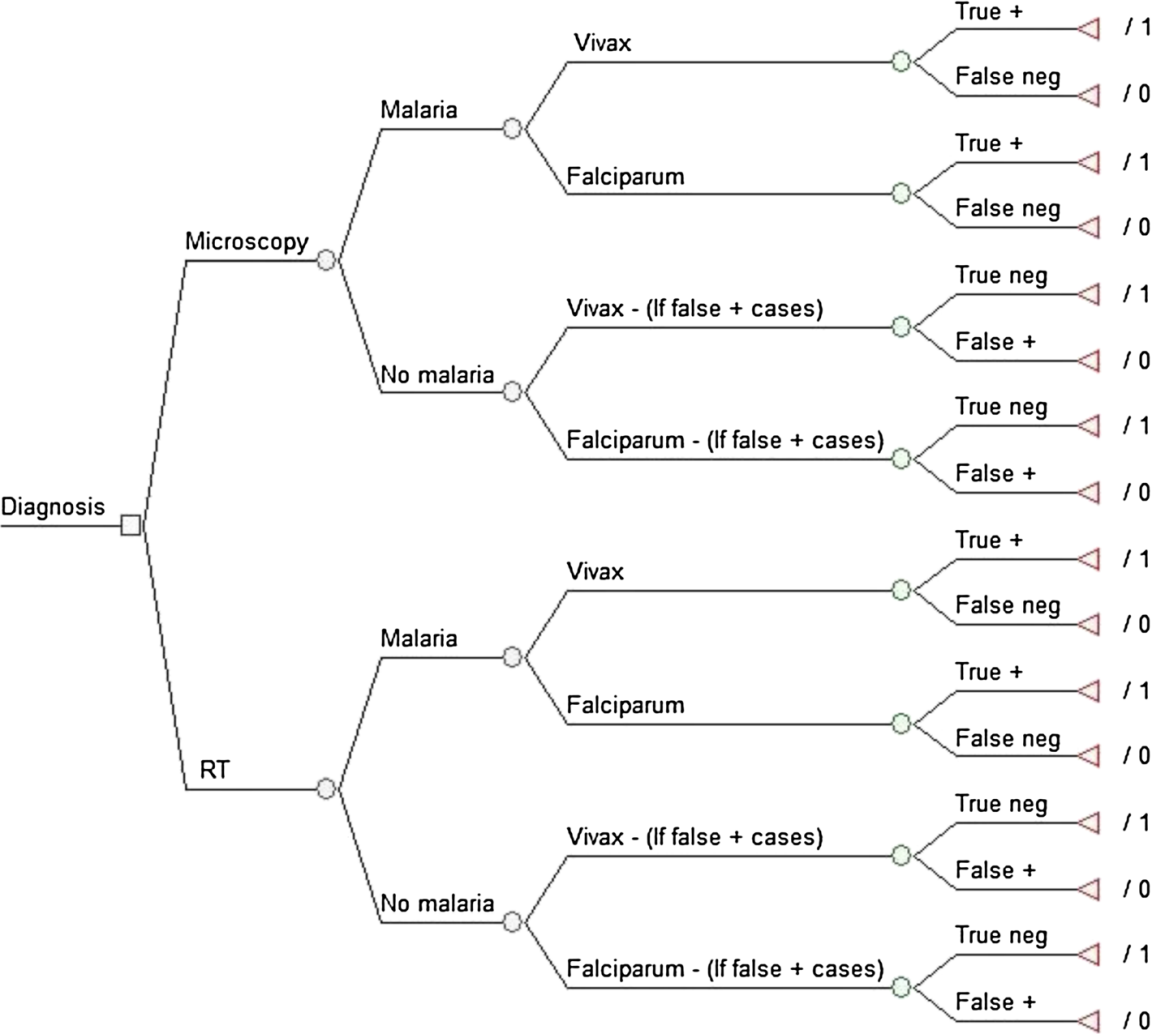
**Basic structure of decision tree for the “adequately diagnosed cases”.** Notes: RT = Rapid Test; Neg = negative; + = positive; /1 = adequately diagnosed case; /0 = inadequate diagnosis.

A decision tree was developed to compare five RDTs with conventional thick smear microscopy as diagnostic strategies for new cases of malaria in the Extra-Amazon Region. Figure 1 presents the basic structure of the decision tree. A hypothetical cohort of all febrile individuals who had a diagnostic procedure for malaria conducted in 2010 in the Extra-Amazon Region was simulated, considering its various probability nodes. All individuals (100%) presenting with fever to health facilities would undergo diagnostic test using either microscopy or one of the five RDTs. They could either have malaria or not, estimated by the prevalence of malaria in the population presenting fever and suspected malaria. If the patient had malaria, the diagnostic test could result positive for malaria, indicating infection due to *P. falciparum* or *P. viv*ax - representing a true positive result (sensitivity); or could result negative (1-sensitivity) representing a false negative result. If the patient did not have malaria, the diagnostic test could result negative, representing a true negative result (specificity); or result positive (1-specificity) representing a false positive result. While true positive and true negative results were considered adequately diagnosed cases, false negatives and false positives were considered incorrectly diagnosed cases. These were the terminal nodes of the decision tree (Figure 1). Cost and epidemiological data were collected and inserted to populate this decision tree.

In the analytical framework, the cases were followed up from fever onset until diagnostic results were provided. The study period was January-December 2010. The analysis perspective was that of the Brazilian Public Health System. The outcome considered in the analysis was adequately diagnosed cases of malaria.

The hypothetical cohort was based on 2,702 valid registered notifications of investigation into malaria in the Extra-Amazon Region, for the year 2010. Ten percent more was added to this number, taken as the wastage rate, making a total of 2,972 diagnostic tests, the number which became the basis of the decision model and of the cost calculations.

### Costs of malaria diagnosis

Only direct diagnostic costs were considered. All costs were calculated in Brazilian Reais and converted to U.S dollars considering the official average exchange rate for 2010 (US$1.00 = R$1.7597) [11].

Cost data were obtained from scientific literature, official government reports and the reimbursement table for procedures by the National Health System. The direct costs of RDTs were obtained from laboratory product suppliers and from the Ministry of Health.

Costs considered for microscopy diagnostic strategy included: thick smear microscopy with consumables, supplies and equipment costs (microscope purchase and maintenance). Costs considered for RDT diagnostic strategies included the direct cost of the RDT and gloves. The RDT costs were varied at 20% above and below the base-case value, to compose the intervals.

Consumables and supply costs for microscopy were aggregated into one estimated cost measure of conducting a single thick smear procedure. In the base-case, the cost of one thick smear procedure was that estimated by Macauley in Brazil [6] based on costs of all used microscopy supplies through a passive case detection diagnosis strategy. Upper level variation for sensitivity analysis also considered Macauley’s [6] estimates through a passive plus active case detection diagnosis strategy. As these costs were estimated for 2001, they were adjusted for inflation considering the Consumer Price Index for 2010 [12]. Lower level variations around the base-case were estimated for sensitivity analysis using micro-costing techniques in which the cost of individual consumables required to perform one thick smear procedure were estimated. Costing method and information sources for micro-costing were published by Oliveira and co-workers (2010) [13] and adjusted for inflation considering the Consumer Price Index [12].

The costs of two consultations in a specialized public outpatient service were included for all strategies, considered the recommended minimum for carrying out the diagnosis.

### Equipment costs

The costs of the microscopes were obtained from the Ministry of Health, based on purchase in 2008, and their maintenance costs were obtained from Oliveira and co-workers (2010) [13]; these costs were also adjusted for inflation considering the Consumer Price Index. It was assumed that there was one microscope available for each professional’s use and an annual maintenance cost for each piece of equipment. The cost of the microscope was averaged over the year based on a 5% depreciation rate and a 15-year average lifespan [14,15].

To estimate the cost of the microscopes and their maintenance, per slide examined, two different costing methods were carried out. The first, called Cost 1, considered the information obtained from the five states that reported 50% of the cases in 2010 (SP, ES, MG, GO and DF) and assumed that a microscope was bought for each reference center for the diagnosis of malaria in that states; the final cost was divided by the number of slides examined and 10% was added for wasted tests. The microscope and maintenances costs per slide in the five states were thus assumed as the standard for the whole Extra-Amazon Region. This form of costing presupposed the use of microscopes exclusively for malaria.

The second costing method, Cost 2, was based on the WHO [16] considering the workload of professionals who diagnose malaria. In this method, costs of equipment and its maintenance were assumed to be shared with other health programmes, and they were estimated using the WHO’s parameters that determine the number of microscopy diagnostic tests performed per hour at four different levels of malaria prevalence [16], considering for the study area the parameters that are estimated for low-prevalence areas. The total costs for each diagnostic strategy for these items represented the average costs in the study area weighted by malaria prevalence.

Costs of salaries, training courses, transportation, quality control procedures, construction and maintenance of laboratories were not considered. Table 1 presents cost components and their respective unit costs for microscopy and the five RDT diagnoses.

**Table 1:** Cost components and unit costs considered for malaria diagnosis

**Items**	**Unit cost for base-case analysis (US$)**	**Unit cost for variation analysis (US$)**	**Information sources**
**Exams and supplies**			
Thick smear – one exam ^(1)^	1.34	0.28 – 2.06	Base-case: Macauley, 2005
			Variation: Macauley, 2005 and de Oliveira *et al.*, 2010, based on IEC/SVS/MS. ^(2)^
SD Bioline FK60 (PF/Pan)™ - one test	0.93	0.74 – 1.12	Base-case: Ministry of Health
			Variation: 20% above and below the base-case.
CareStart (Pan)™ - one test	3.86	3.09 – 4.64	Base-case: Laboratory supplier
			Variation: 20% above and below the base-case
First Response Malaria Combo™ - one test	0.82	0.66 – 0.99	Base-case: Laboratory supplier
			Variation: 20% above and below the base-case
Parascreen™ (Pf/Pan) – one test	0.92	0.74 – 1.10	Base-case: Laboratory supplier
			Variation: 20% above and below the base-case.
BinaxNOW Malaria™ - one test	4.93	3.94 – 5.92	Base-case: Laboratory supplier
			Variation: 20% above and below the base-case.
Latex gloves to use with RDT ^(3)^	0.03	0.07	Base-case and variation: de Oliveira e *et al.*, 2010.
**Health services**			
Consultation in a specialized public outpatient service	11.37	----	National Healthcare System reimbursement table.
**Equipment**			
Microscope – one unit - annual value	432.71^(4)^	292.19^(5)^	Base-case and variation: Ministry of Health.
Microscope maintenance – one annual maintenance	54.10^(6)^	43.53 – 64.92	Base-case and variation: de Oliveira *et al.*, 2010, based on IEC/SVS/MS. ^(2)^
			Variation: 20% above and below the base-case.

Extra-Amazon Region, Brazil, 2010.

Notes:

^(1)^ Corresponds to the individual cost of one test considering the following inputs: glass slide, Giemsa and other dyes.

(all the components for staining), immersion oil, lancet, cotton-wool, alcohol and gloves.

^(2)^ The Malaria Laboratory of the Instituto Evandro Chagas (IEC), of SVS/MS, provided the individual costs of each input for carrying out staining and reading of the slide; costs were calculated for one test produced.

^(3)^ Latex gloves – part of the cost of each RDT.

^(4)^ Annual cost of microscopy for 15 years. The cost of one unit was the basis for obtaining the cost per slide examined, for two different costing methods.

^(5)^ Annual cost of microscopy for 30 years. The cost of one unit was the basis for obtaining the cost per slide examined, for two different costing methods.

^(6)^ The cost of maintenance was the basis for obtaining the cost per slide examined for two different costing methods.

### Epidemiological parameters

The epidemiological parameters included in the analytic model were prevalence of malaria, proportion of malaria cases due to *P. vivax* and *P. falciparum* species, and estimates of sensitivity and specificity of diagnostic techniques for both *P. vivax* and *P. falciparum* infections (Table 2). Secondary sources of epidemiologic data were the scientific literature (Medline, Lilacs and SciElo databases), and the National Malaria Surveillance Information System of the Brazilian Ministry of Health [4].

**Table 2:** Epidemiological parameters considered in the analytic model

**Parameter**	**Base-case value**	**Variation values**	**Information sources**
Prevalence of malaria among febrile patients seeking diagnosis	0.467	0.560 e 0.373	Base-case: Ministry of Health
			Variation: 20% above and below the base-case.
Proportion of malaria cases due to *Plasmodium vivax*	0.713	0.856-0.570	Base-case: Ministry of Health
			Variation: 20% above and below the base-case.
Proportion of malaria cases due to *Plasmodium falciparum*	0.287	0.344-0.230	Base-case: Ministry of Health
			Variation: 20% above and below the base-case.
Sensitivity of microscopy for *Plasmodium vivax*	0.950	0.717	Base-case: Ohrt e *et al.*, 2002 - Variation: Andrade e *et al.*, 2010.
Specificity of microscopy for *Plasmodium vivax*	1.000	0.950	Base-case: Haghdoost *et al.*, 2006; Variation: Haghdoost *et al.*, 2006.
Sensitivity of microscopy for *Plasmodium falciparum*	0.976	0.757	Base-case: Alam *et al.*, 2011; Variation: Andrade *et al.*, 2010.
Specificity of microscopy for *Plasmodium falciparum*	1.000	0.932	Base-case: Andrade *et al.*, 2010; Variation: Alam e *et al.*, 2011;
Sensitivity of CareStart™ for *Plasmodium vivax*	0.953	0.711-1.000	Base-case: Mekonnen *et al.*, 2010; Variation: Upper value: Sharew e *et al.*, 2009. Lower value: Ashley e *et al.*, 2009.
Specificity of CareStart™ for *Plasmodium vivax*	1.000	0.919	Base-case: Mekonnen e *et al.*, 2010; Variation: Ratsimbasoa e *et al.*, 2007.
Sensitivity of CareStart™ for *Plasmodium falciparum*	0.964	0.854-1.000	Base-case: Mekonnen *et al.*, 2010; Variation: Upper value: Sharew e *et al.*, 2009. Lower value: Ashley e *et al.*, 2009.
Specificity of CareStart™ for *Plasmodium falciparum*	1.000	0.881	Base-case: Mekonnen *et al.*, 2010; Variation: Ratsimbasoa e *et al.*, 2007.
Sensitivity of First Response Malaria Combo™ for *Plasmodium vivax*	0.842	0.721-0.925	Base-case: Singh *et al.*, 2010; Variation: Singh *et al.*, 2010
Specificity of First Response Malaria Combo™ for *Plasmodium vivax*	0.940	0.900 – 0.982	Base-case: Bharti e *et al.*, 2008; Variation: Upper value: Singh *et al.*, 2010. Lower value: Bharti *et al.*, 2008.
Sensitivity of First Response Malaria Combo™ for *Plasmodium falciparum*	0.960	0.880-0.990	Base-case: Bharti e *et al.*, 2008; Variation: Bharti e *et al.*, 2008.
Specificity of First Response Malaria Combo™ for *Plasmodium falciparum*	0.950	0.756-0.970	Base-case: Bharti *et al.*,2008; Variation: Upper value: Bharti *et al.*, 2008. Lower value: Singh *et al.*, 2010.
Sensitivity of Parascreen™ for *Plasmodium vivax*	0.772	0.642-0.873	Base-case: Singh e *et al.*, 2010; Variation: Singh e *et al.*, 2010.
Specificity of Parascreen™ for *Plasmodium vivax*	0.981	0.959-0.993	Base-case: Singh *et al.*, 2010; Variation: Singh *et al.*, 2010.
Sensitivity of Parascreen™ for *Plasmodium falciparum*	0.940	0.885-0.974	Base-case: Singh *et al.*, 2010; Variation: Singh *et al.*, 2010.
Specificity of Parascreen™ for *Plasmodium falciparum*	0.720	0.658-0.776	Base-case: Singh *et al.*, 2010; Variation: Singh *et al.*, 2010.
Sensitivity of BinaxNOW™ for *Plasmodium vivax*	0.873	0.729-0.930	Base-case: Wongsrichanalai *et al.*, 2003; Variation: Upper value: Wongsrichanalai *et al.*, 2003. Lower value: Pabón *et al.*, 2007.
Specificity of BinaxNOW™ for *Plasmodium vivax*	0.977	0.995-1.00	Base-case: Wongsrichanalai *et al.*, 2003; Variation: Pabón e *et al.*, 2007.
Sensitivity of BinaxNOW™ for *Plasmodium falciparum*	1.000	0.520-0.960	Base-case: Wongsrichanalai *et al.*, 2003; Variation: Upper value: Farcas *et al.*, 2003. Lower value: Pabón *et al.*, 2007.
Specificity of BinaxNOW™ for *Plasmodium falciparum*	0.962	0.920-0.980	Base-case: Wongsrichanalai *et al.*, 2003; Variation: Wongsrichanalai *et al.*, 2003.
Sensitivity of SD Bioline FK60 (PF/Pan)™ for *Plasmodium vivax*	0.710	0.636(0.424-0.815)	Base-case: Vas Dev, 2004. Variation: Ratsimbasoa, 2008 ^(1)^
Specificity of SD Bioline FK60 (PF/Pan)™ for *Plasmodium vivax*	1.000	0.989(0.946-0.999)	Base-case: Vas Dev, 2004. Variation: Ratsimbasoa, 2008
Sensitivity of SD Bioline FK60 (PF/Pan)™ for *Plasmodium falciparum*	1.000	0.929 (0.889-0.971)	Base-case: Vas Dev, 2004. Variation: Ratsimbasoa, 2008
Specificity of SD Bioline FK60 (PF/Pan)™ for *Plasmodium falciparum*	1.000	0.989 (0.946-0.999)	Caso-base: Vas Dev, 2004. Variação: Ratsimbasoa, 2008

Extra-Amazon Region, Brazil, 2010.

Note: ^(1)^ Exception: The article by Ratsimbasoa (2008) used a combination of PCR and thick smear as the gold standard.

Accuracy studies for the five RDTs were considered only if they used microscopy as the gold standard, and accuracy studies of thick smear microscopy were considered only if they used polymerase chain reaction as the gold standard [17-20]. Quality of published accuracy studies was assessed considering 12 criteria of the Quality Assessment of Diagnostic Accuracy Studies (QUADAS) instrument [21] and three additional criteria judged relevant: socio-demographic characteristics of patients, confidence interval and the sampling method proposed by the Standards for Reporting Studies of Diagnostic Accuracy (STARD) [22].

Fifteen scientific articles were selected as sources for the values used in the base-case and in the variation of accuracy estimates for the diagnostic methods studied (Table 2) [17-20,23-33].

For the epidemiological parameters related to the probability of malaria and its presentation, the intervals of variation were constructed with 20% above and below the base-case value (Table 2).

### Cost, cost-effectiveness and sensitivity analysis

Total costs accrued for malaria diagnosis during the study period were estimated for each diagnostic strategy, considering the total number of tests performed during the study period plus 10% (n = 2.972). Incremental cost-effectiveness ratio (ICER) was calculated considering the incremental cost needed to adequately diagnose one individual with suspected malaria using a RDT as opposed to microscopy. To examine the variability of the cost-effectiveness ratios, one-way sensitivity analysis to investigate the effect of parameter values was conducted. Cost parameters for microscopy and RDT diagnosis were varied, as were malaria prevalence and accuracy estimates for each diagnostic method. Parameter values varied over the upper and lower range estimates. Variation of costs (Table 1) and epidemiologic parameters (Table 2) considered in the analysis and their respective sources of information are presented. TreeAge Pro® software was used to build the decision model and for cost-effectiveness and sensitivity analyses [34].

### Ethical issues

This study was a hypothetical model. It was developed with secondary and not nominal data, not doing experiments in human begins. Conclusions will help to provide benefits to population at risk of malaria transmission.

## Results

Two cost-effectiveness analyses were produced, based on the two costing methods and relative to the costs per microscope test and of the maintenance of microscopes. To identify the analyses, they were labeled “exclusive microscope” – for the Cost 1 method that does not take into account the workload with malaria, assuming that the microscopes are used exclusively to diagnose the disease – and “shared microscope” – for the Cost 2 method that is weighted by the workload of health professionals working with malaria.

The results of cost-effectiveness analysis of the microscopy strategies and of each of the five RDTs analysed, for the outcome “adequately diagnosed cases” with Cost 1 are presented in Table 3 and results with Cost 2 are presented in Table 4. Both analyses (Tables 3 and 4) used the cheapest strategy, the RDT First Response Malaria Combo™, as the baseline with which other strategies were firstly compared. To Cost 1, inicial comparisons of Parascreen, SD Bioline FK60 and CareStart were made to First Response Malaria Combo, the cheapest strategy; ICT BinaxNOW and Microscopy were compared to CareStart conforming sequence presented in Table 3. To Cost 2, inicial comparisons of Parascreen, SD Bioline FK60 and Microscopy were made to First Response Malaria Combo, the cheapest strategy; CareStart ICT and BinaxNOW were compared to Microscopy conforming sequence presented in Table 4.

**Table 3:** Results of the cost-effectiveness analysis of strategies for malaria diagnosis with exclusive-use microscopy, per adequately diagnosed case

**Strategies for malaria diagnosis**^**(1)**^	**Cost per case (US$)**	**Additional cost (US$)**	**Effect**	**Additional effect**	**Incremental cost-effectiveness ratio (US$)**
First Response Malaria Combo	12.22	____	0.9116		____
Parascreen	12.32	0.10	0.8660	−0.0456	(Dominated)
SD Bioline FK60	12.33	0.11	0.9034	−0.0082	(Dominated)
CareStart	15.26	3.04	0.9795	0.0679	**44.77**
ICT BinaxNOW	16.33	1.07	0.9432	−0.0363	(Dominated)
Microscopy	36.59	21.33	0.9801	0.0006	**35,550.00**

Extra-Amazon Region, Brazil, 2010.

Note: ^**(1)**^ Inicial comparisons of Parascreen, SD Bioline FK60 and CareStart were made with First Response Malaria Combo, the cheapest strategy. ICT BinaxNOW and Microscopy were compared to CareStart.

**Table 4:** Results of the cost-effectiveness analysis of strategies for malaria diagnosis with shared-use microscopy, per adequately diagnosed case

**Strategies for malaria diagnosis**	**Cost per case (US$)**	**Additional cost (US$)**	**Effect**	**Additional effect**	**Incremental cost-effectiveness ratio (US$)**
First Response Malaria Combo	12.22	____	0.9116		____
Parascreen	12.32	0.10	0.8660	−0.0456	(Dominated)
SD Bioline FK60	12.33	0.11	0.9034	−0.0082	(Dominated)
Microscopy	12.77	0.55	0.9801	0.0685	**8.03**
CareStart	15.26	2.49	0.9795	−0.0006	(Dominated)
ICT BinaxNOW	16.33	3.56	0.9432	−0.0363	(Dominated)

Extra-Amazon Region, Brazil, 2010.

Note: ^**(1)**^ Inicial comparisons of Parascreen, SD Bioline FK60 and Microscopy were made with First Response Malaria Combo, the cheapest strategy. CareStart and ICT BinaxNOW Microscopy were compared to Microscopy.

In the results shown in Table 3, the tests Parascreen™ and SD Bioline FK60™ were dominated by the First Response Malaria Combo™. The ICT BinaxNOW™ test was dominated by CareStart™. The RDT CareStart™ was the most cost-effective diagnostic strategy. Microscopy was the most expensive and most effective, with an additional case adequately diagnosed by microscopy costing US$ 35,550.00 in relation to CareStart™.

When the cost of microscopes and their maintenance was used weighted by workload with malaria (cost 2), the base-case result is exactly the opposite, making microscopy an extremely cost-effective strategy compared to First Response Malaria Combo™. All the other RDTs were dominated (Table 4).

### Sensitivity analysis

In sensitivity analysis, cost-effectiveness ratios were sensitive to few parameters. Using basic costs of RDT, only two technologies - ICT BinaxNOW™ and CareStart™- have a higher individual cost than the cost estimated for microscopy, the standard technology used in the public health system. One-way sensitivity analysis of the costs of each of these two RDT was carried out for the two analytic models. Both for the analytic model with exclusive use of microscopy (Cost 1) and for the shared microscopy (Cost 2) the reduction of 20% in the cost of each of the cited tests, in accordance with the intervals considered, did not alter the results for the base-case.

In the one-way sensitivity analysis relative to the model with exclusive microscopy (Cost 1 – Table 3), when the pre-test probability (prevalence) was reduced to 37.30%, the RDT SD Bioline™ became the most cost-effective strategy, with ICER of US$ 21.59 per adequately diagnosed case, in comparison with First Response Malaria Combo™.

In the sensitivity analysis for the shared microscope model (Cost 2), microscopy continued to be cost-effective, even with reductions in its accuracy (Table 5), according to the values from the sources, except when the sensitivity to *P. vivax* varied to 0.7170; in this situation it was dominated by First Response Malaria Combo™ (Table 5). At the same model, when the estimated sensitivity for SD Bioline FK60™ for *P.vivax* reached the maximum value found in the scientific literature - 81.50% - , this RDT became extremely cost-effective, in relation to the cheapest strategy, with an ICER of only US$ 3.98 per adequately diagnosed case. In the same analysis, microscopy was also cost-effective compared to SD Bioline, with an ICER of US$ 10.62 per adequately diagnosed case.

**Table 5:** Results of the one-way sensitivity analysis for the model with shared-use microscopy, considering variations in the accuracy of the microscopy

Parameter * Accuracy of microscopy	**Variation value**	**Incremental cost-effectiveness ratio (US$) of microscopy compared to First Response Malaria Combo**
Sensibilidade – *P. vivax*	0.7170	(Dominated)
	0.8585	14.47
Sensibilidade – *P. falciparum*	0.7570	14.06
	0.8785	9.93
Especificidade – *P. vivax*	0.9500	11.12
Especificidade – *P. falciparum*	0.0320	9.48

Extra-Amazon Region, Brazil, 2010.

Note: * Only variation values in which changes in ICER occurred are presented.

Introducing a “proxy” measurement into the analytic model with shared microscopy (Cost 2) for individual access to the diagnosis, assuming a probability of 100% of access for a public health system user to the RDT and, hypothetically, of 85% of access to microscopy – due to its technical requirements and the relocation needed to reach places where the test is carried out – microscopy saw its effectiveness reduced and was dominated by the RDT CareStart™. CareStart™, in this variation, presented ICER per diagnosed case of US$ 44.75 compared to the cheapest strategy.

## Discussion

A review of 55 studies of the costs and 43 studies of the cost-effectiveness of malaria interventions revealed that most studies dealing with economic evaluations for malaria diagnostic are carried out in Africa, with only 4% of them taking place in South America [35]. This is the first cost-effectiveness study in Brazil that evaluated more than one commercial RDT brand simultaneously, and the first that took place outside the Amazon Region.

When making informed decisions on RDT introduction, as recommended for the Brazilian Extra-Amazon Region, a product with high accuracy should be selected, as recommended by the WHO [8]. Although various different RDTs are available, they can differ significantly in sensitivity and specificity values.

In Brazil, there are two published economic evaluation studies of malaria diagnosis; both were conducted in the Amazon Region [7,13]. The first was a cost-minimization analysis conducted in rural Brazil, which demonstrated that the RDT ParaSight-F® was more cost-effective than microscopy, mainly due to significantly lower transportation costs when using RDTs [7].

The second Brazilian study was by Oliveira and co-workers [13] who evaluated a commercial RDT brand, OptiMal®, in Amazonian areas not covered by a microscope-equipped laboratory. The study concluded that the RDT would be more cost-effective in these areas if the accuracy of microscopy practiced in the field was lower than values assumed in the base-case.

Lubell and co-workers [36] and Chanda and co-workers [37] also demonstrated that RDT was cost-effective when compared to microscopy, in African countries, in settings where microscopy accuracy was low. Lubell and co-workers [36], Chanda and co-workers [37], Shillcutt and co-workers [38], and Rolland and co-workers [39] pointed to the need for alternative malaria diagnostic methods in studies conducted in Africa, where low accuracy of diagnosis was observed.

In recent years, new cost-effectiveness studies for malaria diagnosis have been carried out in African countries. The differing epidemiological situations and various methodologies used in these studies prevent further comparisons with the current evaluation, but it can be noted that the published African studies concluded that RDTs are more cost-effective in high and low transmission situations in Uganda [40], in association with the use of artemisinin-based drugs in Senegal [41] in areas of Mozambique [42] and of Ethiopia [43] when compared to presumptive treatment or to microscopy. In the African situations, the use of RDTs leads to rational use of medication, avoiding the waste that can occur with presumptive treatment.

In the present study, when assuming the use of microscopy laboratories exclusively for malaria diagnosis, this strategy was not cost-effective, since an additional microscopy-diagnosed case was estimated at over 35,000 dollars comparing to CareStart™, due to the vast difference in the cost of microscopy compared to other strategies if equipment is only used for malaria diagnosis. It should be noted that this is more than three times the Brazilian *per capita* Gross Domestic Product for the year 2010, which was US$ 10,806.39 [44]. Due to the low number of tests run in the Extra-Amazon area, exclusive use of equipment and structures for thick smear test, not sharing with other programmes, would be the worst possible use of resources. However, in the cost method that weighted malaria workload for the professionals in the Extra-Amazon area, microscopy presented a fairly rational use of resources, due to its low cost and high effectiveness.

It is important to stress that in analyses presented in Tables 3 and 4, the supposition was that the strategies would use the same health service organization. Thus, without considering the possibility of expanded access to diagnosis with the introduction of a RDT, and assuming that microscopy equipment was shared with other health programmes, the thick smear test would be the most cost-effective strategy (Table 4). If public health services provided easier access to RDTs in the Brazilian states of the Extra-Amazon Region, differentiating the probability of access to RDTs from access to microscopy, then the RDT CareStart™ would be more cost-effective, even assuming shared use of microscopy with other programmes and maintaining assumptions of the model under study.

SD Bioline FK60™ is the RDT technology that the Brazilian Ministry of Health has bought in recent years. The search for data on its accuracy, however, was not very efficient. Various RDTs are produced by the Bioline laboratory, and there are few scientific articles that clarify the use of the same test used in Brazil: the FK 60 with enzymes HRP2 and pLDH. As a RDT used in Brazil, it should be thoroughly evaluated in terms of its accuracy, so that the country has its own set of valid results that can be applied in cost-effectiveness analyses and in other studies that aid decision-making. It would also be very interesting and helpful to know the accuracy levels of microscopy practiced in the Extra-Amazon Region, especially considering the low number of slides examined, which can contribute to loss of experience in the diagnostic exam.

It’s important to note that, differently from Amazon Region, the diagnostic in Extra-Amazon Region, either with rapid tests or microscopy, involves the same professionals in its execution. Thus there are not relevant differences in other costs as salaries or transportation, being the cost of equipments and its maintenance and the direct cost of exams the most important differences between the strategies. The analysis didn’t extend to treatment of patients because of the lacking information about the treatment of non-malaria cases, which prevents a complete analysis of the economic consequences of misclassified cases and because of the greater interest in the costs of diagnosis itself to help decisions about the use of rapid tests in Brazil. As it has been noted in other settings of malaria transmission [38], improving data on treatment of non malaria cases in Brazil is urgently needed to allow the expansion of economic analysis for this disease.

## Conclusion

The analysis of cost-effectiveness of malaria diagnosis technologies in the Brazilian Extra-Amazon Region depends on the exclusive or shared use of the “microscopy” strategy. Following the assumptions of this study, shared microscopy would be the most cost-effective strategy of the six technologies evaluated. However, if used exclusively for diagnosing malaria, microscopy would be the worst use of resources. Access to the diagnosis is also an important factor in evaluating cost-effectiveness of strategies. Microscopy would not be the most cost-effective strategy, even when structure is shared with other programmes, when the probability of a patient having access to it was reduced and the probability of having access to RDTs was 100%. Under these circumstances, the RDT CareStart™ would be the most cost-effective strategy. This discussion is very important for managers to consider if RDTs are introduced into these areas to broaden access to diagnosis.

## Competing interests

The authors declare that they have no competing interests.

## Authors’ contributions

MRFO conceived the study, participated in the design of the study, performed the analysis and drafted the manuscript. SPG conceived the study and participated in its analysis. HMP conceived the study and participated in its analysis. GASR coordinated the study and participated in its design and analysis. All authors read and approved the final manuscript.
